# Sex Differences in Primary Mitral Regurgitation Assessment: Highlighting the Role of Regurgitant Fraction

**DOI:** 10.1016/j.jacadv.2024.101020

**Published:** 2024-06-03

**Authors:** Ana G. Almeida

**Affiliations:** Heart and Vessels Department, Cardiology, University Hospital Santa Maria, Faculty of Medicine of Lisbon University, CCUL@RISE, CAML, Lisbon, Portugal

**Keywords:** mitral regurgitation, magnetic resonance, regurgitant fraction

**Figure:**
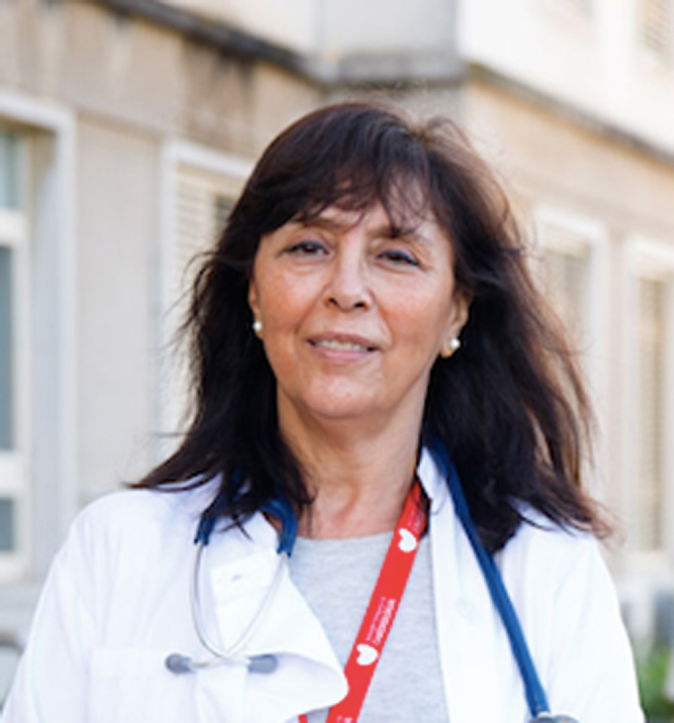

Sex differences, regarding the assessment of mitral regurgitation (MR) severity and cardiac remodeling, have been scarcely addressed and notably absent in current recommendations. In fact, current guidelines propose variable cut-offs that are applied uniformly to both sexes. Studies are scarce on this regard since, in most studies, women have been under-represented and sex has not been taken into account.^1^^,^^2^

However, recent studies^3^^,^^4^ have suggested that women have a delayed referral for mitral valve intervention, despite more symptomatic, with evidence of more compromised left ventricle (LV) function and likely increase in post-operative risk. This may occur because MR is more frequently diagnosed as moderate in women in spite of symptoms, and additionally, LV dysfunction and dilatation are often misdiagnosed, namely when using values without normalization for body surface area.^5^ The assessment of the MR severity in women and the differences between sexes are challenging and need further focused analysis.

For acknowledging differences between sexes in regard to primary MR assessment and its impact on indication for repair, it is crucial not only to identify differences in severity criteria and to define cut-offs for proposing timely management but also to understand the pathophysiology behind those differences.

Women with moderate to severe and severe MR were found to have significantly smaller LV and stroke volumes than men.^6^ Also, in patients with organic MR,^7^^,^^8^ women in comparison with men were found to have smaller end-diastolic and end-systolic LV dimensions and lower regurgitant volume. However, these findings were related with the features of advanced disease, such as higher pulmonary pressure, more atrial fibrillation, and heart failure symptoms. The ensuing valve surgery is often delayed by the classification of MR as moderate as based in conventional cut-offs for regurgitant volumes.

In the current study,^9^ the authors aimed to evaluate the phenotypes of primary MR by mitral valve prolapse, according to sexes. Additionally, they aimed comparing men and women regarding the relationship between regurgitation severity and cardiac remodeling with hallmarks of advanced disease such as functional class, LV dilatation, left atrial (LA) dilatation, and pulmonary hypertension. In a large cohort of patients with moderate to severe and severe MR due to valve prolapse referred for MV intervention, the authors analyzed retrospectively data from patients that underwent both echocardiography and cardiovascular magnetic resonance (CMR) for MR severity and cardiac remodeling assessment. In this cohort, women were older than men, had higher NYHA functional class, and larger indexed LA volumes, all hallmarks of clinical severity, despite showing lower MR effective regurgitant orifice area (EROA), regurgitant volumes, as well as ventricular volumes than men. The optimal threshold values for the regurgitant volume and EROA associated with abnormally increased LV size (according to reference) were consistently lower in women than in men. Moreover, for the same regurgitant fraction, regurgitant volumes in women were significantly lower. Regurgitant fraction, in contrast to regurgitant volume, was consistently associated with clinical adverse manifestations such as NYHA functional class III/IV, severe LA dilatation, or pulmonary hypertension, in both sexes. Importantly, optimal cut-offs for regurgitant fraction regarding clinical adverse features were similar in both sexes, while regurgitant volumes were different according to sex even when indexed to body surface area.

A previous study by House et al^10^ using CMR described that a regurgitant fraction of 40% correlated with different regurgitant volumes according to sex, with smaller volumes in women, suggesting a gender-independent value for the regurgitant volume in the assessment for MR. In the current study, Altes et al go further, showing that regurgitant fraction had a consistent association with the hallmarks of adverse clinical outcomes with similar cutoffs in both sexes, in contrast with RV even after indexing to body surface area.

The authors discuss elegantly the findings and raise appropriate hypothesis for justifying the pathophysiology underlying the smaller regurgitant volume in women even when indexed to body surface area. It is conceivable that a more restrictive physiology in women probably related to the smaller heart size and a more advanced myocardial disease, with more fibrosis in advanced age, may have had impact on the LV volumes and cut-offs of severity using this parameter. A recent study on organic MR referred for intervention^11^; women presented evidence of more raised LA stiffness than men, suggesting more advanced cardiac disease. On the other side, indexing the regurgitant volume to the LF total stroke volume seems to provide a more robust index that also takes into account the LV size, providing a unified parameter for severity assessment.

This study represents in advance in knowledge regarding sex-related differences in the clinical and imaging phenotypes of primary MR using state-of-art imaging modalities like CMR and echocardiography. This suggests that using a single EROA or regurgitant volume cut-off values as recommended for grading MR severity in women would place MR severity in the range of moderate, despite the underestimate on the impact of the LV remodeling and delay the referral to mitral valve repair.

Further prospective studies should be undertaken for confirmation of findings, which are promising and with substantial support from an appropriate design and robust methods. First, lower regurgitant volumes and EROA cut-offs for MR severity may be more appropriate from the pathophysiological point of view. Second, regurgitant fraction seems a more robust and independent parameter of severity for both sexes. As also suggested by the authors, this parameter should also be assessed against long-term follow-up for appropriate prognostic purposes and better therapeutic decisions.

Of note, an important issue regards the limitation for echocardiography in challenging cases of primary mitral valve such as late systolic MR or multiple jets in organic MR where concordance between echo and CMR is poor and where regurgitant volume by CMR has been shown to be stronger predictor for mortality or indication for surgery in comparison to echo.^12^

The authors should be congratulated for this nicely designed study that explores the assessment of severity of primary MR in women, a recently identified gap in knowledge that opens new avenues for increased precision in the diagnosis of this valve heart disease.

## Funding support and author disclosure

The author has reported that she has no relationships relevant to the contents of this paper to disclose.
